# 

**Published:** 1860-04

**Authors:** 


### PENNSYLVANIA COLLEGE OF DENTAL SURGERY

SESSION 1859-'60.

### FACULTY.

T. L. BUCKINGHAM, D. D. S.,
Prof. of Chemistry and Metallurgy.

J. H. McQUILLEN, D. D. S.,
Prof. of Anatomy and Physiology.

WILLIAM CALVERT, D. D. S.,
Prof. of Mechanical Dentistry.

C. N. PIERCE, D. D. S.,
Prof. of Dental Physiology and Operative Dentistry.

J. L. SUESSEROTT, D. D. S.,
Prof. of the Principles of Dental Surgery and Therapeutics.

D. H. GOODWILLIE, D. D. S.,
Demonstrator of Operative Dentistry.

J. J. GRIFFITH, D. D. S.,
Demonstrator of Mechanical Dentistry.

The regular Course will commence on the first Monday of November, and continue until the first of March ensuing.

During October, the Laboratory will be open, and a Clinical Lecture delivered every Saturday by one of the Professors, at 3 o'clock P. M.

The most ample facilities are furnished for a thorough course of practical instruction.

Tickets for the Course, Demonstrators' Tickets included, $100; Matriculation Fee $5; Diploma Fee $30. For further information, address W. CALVERT, DEAN,
133 North Eleventh Street, PHILADELPHIA.

---

### OSTEOPLASTIC.

Dr. Pearson, in offering the Osteoplastic, does so with full confidence that it will be received with a hearty welcome by all liberal minded men of the profession.

The Osteoplastic has been submitted to a thorough test of more than two years in the teeth of various patients, as well as those of the discoverer himself, before announcing it to th profession.

The Osteoplastic has many advantages over *even gold*, being in color much the same as the teeth; it also adheres firmly to the bone, consequently, is not liable to come out of ill-shaped cavities. As it is introduced in a plastic state, it saves much time and trouble, and requiring little or no pressure, the most delicate front teeth can be perfectly filled and restored to their original beauty, no matter how badly decayed, if not otherwise diseased.

The Osteoplastic never changes color or discolors the tooth, hence, its peculiar adaptation for front teeth, especially those of a white, delicate structure.

Another great advantage the Osteoplastic possesses over gold for filling is in the fact of its being a non-conductor, no after pain is experienced by the patient from hot or cold drinks, as is often the case with gold or other fillings

The Osteoplastic hardens in from three to five minutes, so as not to be disturbed by rinsing the mouth, and, in a few hours, so as to resist the action of mastication.

In a word, no reasonable objections can be urged against it by the honest and intelligent members of the profession.

The Osteoplastic is now manufactured and offered for sale by the discoverer at $5 for 1 oz. packges, and $3 for ½ oz. packages.

It can be sent to any part of the United States without risk, being neatly and securely pu up in boxes with full instructions &c.

Orders, through JONES, WHITE & McCURDY of New York, J. T. TOLAND of Cincinnati, directed to the Subscriber, will meet with prompt attention.

JAMES PEARSON, Dentist,
**No. 1085 Broadway, Cor. West 31st Street.**

New York, Septem 1, 1859,

### BALTIMORE COLLEGE OF DENTAL SURGERY.

**TWENTY-FIRST ANNUAL SESSION 1860-61.**

**FACULTY.**

CHAPIN A. HARRIS, D. D. S., M. D.
*Principles of Dental Science and Practice of Dental Surgery.*

THOMAS E. BOND, M. D.
*Therapeutics and Materia Medica.*

PHILIP H. AUSTEN, D. D. S., M. D.
*Mechanical Dentistry.*

REGINALD N. WRIGHT, M. D.
*Chemistry and Metallurgy.*

A. SNOWDEN PIGGOT, M. D.
*Anatomy and Physiology.*

CHRISTOPHER JOHNSTON, M. D.
*Microscopical and Comparative Anatomy of the teeth.*

FERDINAND J. S. GORGAS, D. D. S.,
*Demonstrator of Mechanical Dentistry.*

SAMUEL T. CHURCH, M. D., D. D. S.
*Demonstrator of Operative Dentistry.*

The twenty-first Annual Session will commence on the 1st. day of October, and close on the 1st of March.

The regular course of LECTURES will commence on the 1st of November.

The entire day during October, and the afternoon of each day during the rest of the session, will be devoted to Practical Dentistry and Anatomical Dissections.

The Infirmary has a large and rapidly increasing supply of patients, affording to the student unsurpassed facilities for the acquirement of a PRACTICAL knowledge of every Operation in Surgical Medical and Mechanical Dentistry.

Professors' Tickets, $100: Demonstrators' Tickets, $20; Matriculation Fee, $5; Diploma Fee, $30. Dissection Ticket, (optional), $10.

For further information, address **P. H. AUSTEN**, DEAN OF THE FACULTY,
79 North Charles Street, Baltimore.

---

**PUBLISHED MONTHLY.**

### THE DENTAL REVIEW,

**A FIRST CLASS PUBLICATION,**

DEVOTED TO THE INTERESTS OF THE DENTAL PROFESSION.

THE REVIEW contains leading and other original articles on the practice and science of the profession.

Full reports of all important proceedings at the College of Dentists, and general information on Dental matters, in Europe and America.

London, Boilliere, 219 Regent St.; New York, Boilliere, 290 Broadway.

PHILADELPHIA:

LINDSAY & BLAKISTON.

### THIRTEENTH VOLUME

OF THE

### DENTAL REGISTER OF THE WEST,

EDITED BY

**J. TAFT and GEO. WATT.**

**Issued Monthly. - - - $3 a year in Advance.**

The Volume commences in July. CONTRIBUTIONS to the pages of the Register respectfully solicited.

Exchanges and Correspondents will please address "Dental Register," or J. TAFT, Cincinnati, O; Business communications to JNO. T. TOLAND.

The Register being issued Monthly is always fresh, and in advance of all others, therefore indispensable to the practitioner who desires to keep posted up with the new inventions and improvements which are daily developed.

Neither expense nor effort will be spared to give value and variety to its contents. Articles will be illustrated with well executed engravings, whenever they will add to the interest or assist in explanation.

Its extensive circulation and frequency of issue makes it the BEST DENTAL ADVERTISING MEDIUM in the United States.

### TERMS OF ADVERTISING.

| | One Year. | 6 Mo. | 3 Mo. | 1 Insertion. |
|---|---|---|---|---|
| One Page | $50 | $30 | $20 | $15 |
| Half | 30 | 20 | 15 | 10 |
| Quarter | 20 | 15 | 10 | 7 |
| One-Eighth | 15 | 10 | 7 | 5 |

**JNO. T. TOLAND, Pub. and Proprietor,**

38 *West Fourth Street, Cincinnati, Ohio.*

